# Artificial Intelligence Model for Antiinterference Cataract Automatic Diagnosis: A Diagnostic Accuracy Study

**DOI:** 10.3389/fcell.2022.906042

**Published:** 2022-07-22

**Authors:** Xing Wu, Di Xu, Tong Ma, Zhao Hui Li, Zi Ye, Fei Wang, Xiang Yang Gao, Bin Wang, Yu Zhong Chen, Zhao Hui Wang, Ji Li Chen, Yun Tao Hu, Zong Yuan Ge, Da Jiang Wang, Qiang Zeng

**Affiliations:** ^1^ Senior Department of Ophthalmology, The Third Medical Center of Chinese PLA General Hospital, Beijing, China; ^2^ Beijing Airdoc Technology Co., Ltd., Beijing, China; ^3^ Health Management Institute, The Second Medical Center & National Clinical Research Center for Geriatric Diseases, Chinese PLA General Hospital, Beijing, China; ^4^ IKang Guobin Healthcare Group Co., Ltd., Beijing, China; ^5^ Department of Ophthalmology, Shanghai Shibei Hospital of Jing’an District, Shanghai, China; ^6^ Department of Ophthalmology, Beijing Tisnghua Changgung Hospital, Beijing, China

**Keywords:** cataract, artificial intelligence, auxiliary diagnosis, fundus image, convolution neural network

## Abstract

**Background:** Cataract is the leading cause of blindness worldwide. In order to achieve large-scale cataract screening and remarkable performance, several studies have applied artificial intelligence (AI) to cataract detection based on fundus images. However, the fundus images they used are original from normal optical circumstances, which is less impractical due to the existence of poor-quality fundus images for inappropriate optical conditions in actual scenarios. Furthermore, these poor-quality images are easily mistaken as cataracts because both show fuzzy imaging characteristics, which may decline the performance of cataract detection. Therefore, we aimed to develop and validate an antiinterference AI model for rapid and efficient diagnosis based on fundus images.

**Materials and Methods:** The datasets (including both cataract and noncataract labels) were derived from the Chinese PLA general hospital. The antiinterference AI model consisted of two AI submodules, a quality recognition model for cataract labeling and a convolutional neural networks-based model for cataract classification. The quality recognition model was performed to distinguish poor-quality images from normal-quality images and further generate the pseudo labels related to image quality for noncataract. Through this, the original binary-class label (cataract and noncataract) was adjusted to three categories (cataract, noncataract with normal-quality images, and noncataract with poor-quality images), which could be used to guide the model to distinguish cataract from suspected cataract fundus images. In the cataract classification stage, the convolutional-neural-network-based model was proposed to classify cataracts based on the label of the previous stage. The performance of the model was internally validated and externally tested in real-world settings, and the evaluation indicators included area under the receiver operating curve (AUC), accuracy (ACC), sensitivity (SEN), and specificity (SPE).

**Results:** In the internal and external validation, the antiinterference AI model showed robust performance in cataract diagnosis (three classifications with AUCs >91%, ACCs >84%, SENs >71%, and SPEs >89%). Compared with the model that was trained on the binary-class label, the antiinterference cataract model improved its performance by 10%.

**Conclusion:** We proposed an efficient antiinterference AI model for cataract diagnosis, which could achieve accurate cataract screening even with the interference of poor-quality images and help the government formulate a more accurate aid policy.

## Introduction

Cataracts are the leading cause of blindness worldwide (Flaxman et al., 2017). According to the etiological classification, the most common type is age-related cataracts (Tang et al., 2016). In China, the incidence rate of cataracts is as high as 80% in 60–89-year-old people and is almost 90% in the elderly over 90 years old (National Health Commission, 2020). With the acceleration of population aging, the prevalence of cataracts is expected to increase (Song et al., 2018). Early diagnosis and timely surgery can effectively treat cataracts to improve the vision and quality of life of patients (Limwattananon et al., 2018; Wu et al., 2019). However, due to the uneven distribution of medical resources, the shortage of ophthalmologists, and the increase in the number of cataract patients, many cataract patients cannot receive early diagnosis and effective treatment, particularly in the primary medical facilities of low- and middle-income countries (Ramke et al., 2017).

At present, slit lamp camera images are widely applied for cataract diagnosis due to their optical feature and legibility (Zhang et al., 2019). However, there are some limitations to a certain degree in rural areas, i.e., the nonportability of slit lamp devices and the shortage of medical device technicians. In comparison, fundus photographs have several advantages in their efficiency and their handleability. Meanwhile, with the universal application of artificial intelligence (AI) for disease diagnosis, some work focus on automatic cataract detection using AI technique (Patel et al., 2009; Castaneda et al., 2015; Harini and Bhanumathi, 2016; Long et al., 2017). Therefore, combining fundus images and AI-based methods is regarded as a more feasible scheme for automatic cataract detection in actual applications (Park et al., 2020).

Several studies work on AI-assisted diagnosis models of cataracts based on fundus images. Li et al. (2010) divided the fundus images into normal and abnormal, which was an earlier method to apply machine learning to fundus image classification. Xu et al. (2020) proposed a deep learning approach to integrate global and local cataract features to construct a hybrid global–local feature representation model. Ran et al. (2018) extracted the initial feature using deep convolutional neural networks (CNNs) and detected the level of cataracts through random forests. Yang et al. (2016) proposed an ensemble learning-based method to improve the accuracy of cataract diagnosis. Triyadi et al. (2022) processed the two-class cataract classification using VGG-19, Resnet-50, and Resnet-100, whereas several studies improved the cataract classification into four categories, including normal, immature, mature, and hyper mature using the hybrid model (Imran et al., 2020; Simanjuntak et al., 2022). In the study, 1239 fundus images were used to train the model, and three independent feature sets (i.e., wavelet-, sketch-, and texture-based features) were extracted from each fundus image. Two learning models were established, and then, the ensemble method combining the double models was used to classify the fundus image. The best performance of the ensemble method for cataract classification and four-level grading tasks was 93.2% and 84.5%, respectively. Zhang et al. (2019) proposed a six-level cataract grading method that focused on multifeature fusion based on stacking. They extracted two kinds of features that can distinguish the level of cataracts from 1352 fundus images and created a frame consisting of two supported vector machine classifiers and a fully connected neural network to grade cataracts. The average accuracy of the six-level grading model was 92.66%. Previous studies have focused on the use of AI for the identification and grading of age-related cataracts based on normal-quality fundus images, and they are less likely to consider the quality of fundus images (Gao et al., 2015; Guo et al., 2015; Dong et al., 2017).

**FIGURE 1:**
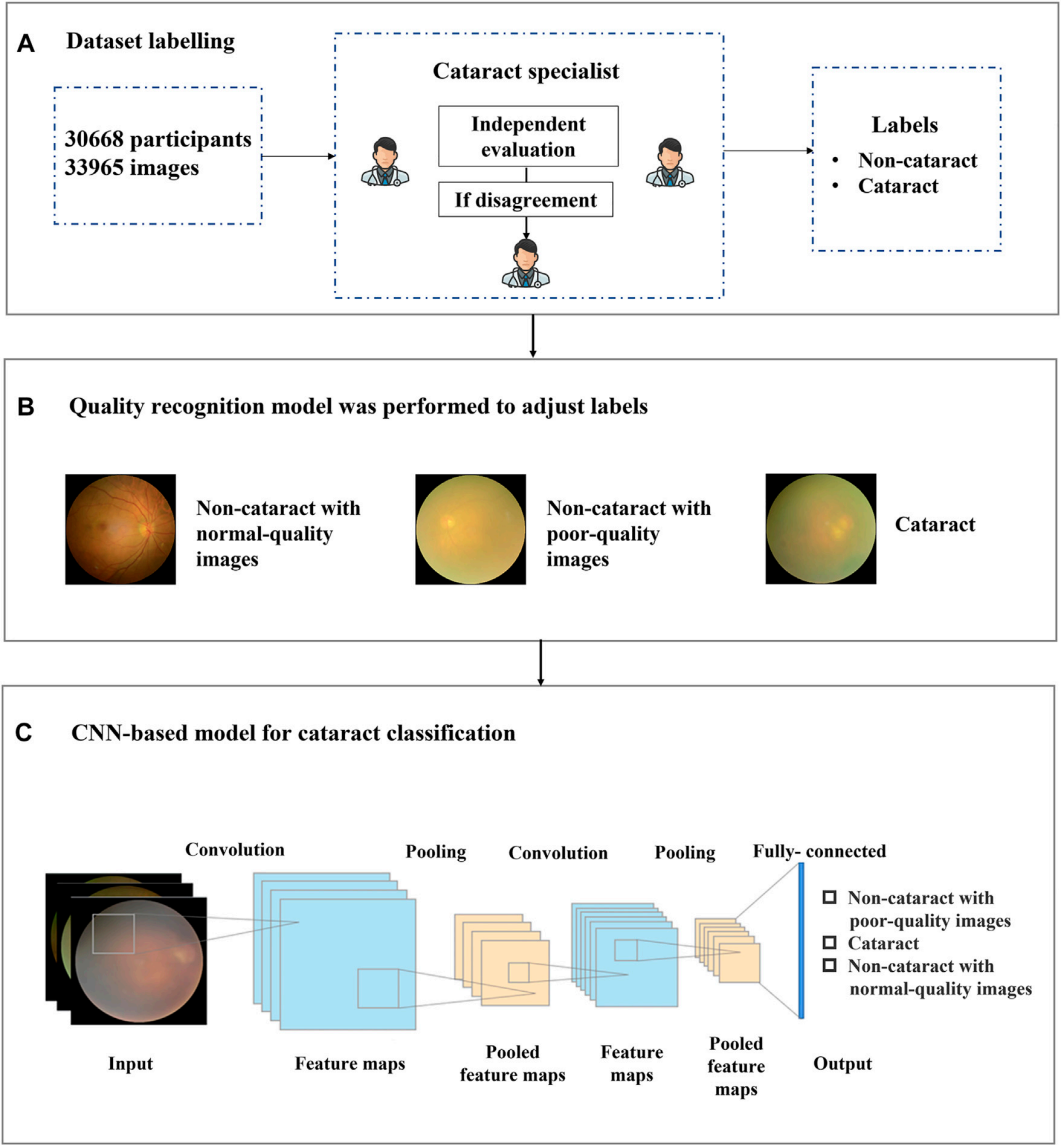
Overall training pipeline for the cataract artificial intelligence model. **(A)** the dataset included 33,965 images of 30,668 participants. Each image was independently labeled by two experienced ophthalmologists, and a third ophthalmologist was consulted if a disagreement arose between the initial ophthalmologists. **(B)** all 33,965 images with binary-class diagnosis labels were adjusted and reassigned to three categories of labels by the quality recognition model. **(C)** all 33,965 images were input to the convolutional neural networks-based model for training and validating the antiinterference cataract artificial intelligence classification model.

However, for cataract detection, the issue of image quality must be considered in actual scenarios since the existence of poor-quality fundus images caused by inappropriate optical scenes in the real world is likely to be mistaken as cataract images, which may cause performance degradation and false positives of cataract diagnosis to some extent. Figure 1 showed the typical noncataract with poor-quality image, noncataract with normal-quality image, and cataract image, respectively. The figure shows that noncataract with poor-quality image is easily mistaken as cataract, which will bring some challenges to cataract identification. In the study, the criteria to distinguish normal and poor-quality noncataract images mainly depended on the lighting and exposure of fundus images. For normal noncataract images, they were under moderate exposure, in which the junction between the rim of the optic disk and the optic cup, the small blood vessels on the surface of the optic disk, and the normal retinal nerve fiber layer were clearly distinguishable as a reference. As for poor-quality noncataract images, there were two main manifestations, including underexposure and light leaking. The first type was that fundus images were generally blurred and dark due to underexposure. In addition, the other one showed a yellow edge, a light leakage-like edge, or a water drop-like reflective band of the surrounding area of the fundus image.

Therefore, in our study, to alleviate the issue of diagnostic performance degradation due to the interference of poor-quality fundus images, we proposed a hybrid structure based on a two-stage AI model to achieve accurate cataract image recognition with the distraction of image quality. The results show that our proposed method has increased the robustness of the model and achieved accurate cataract detection even with numerous interferences. Furthermore, it can assist doctors in cataract diagnosis more efficiently.

## Materials and Methods

### Dataset Collection and Labeling for Artificial Intelligence Model

**FIGURE 2:**
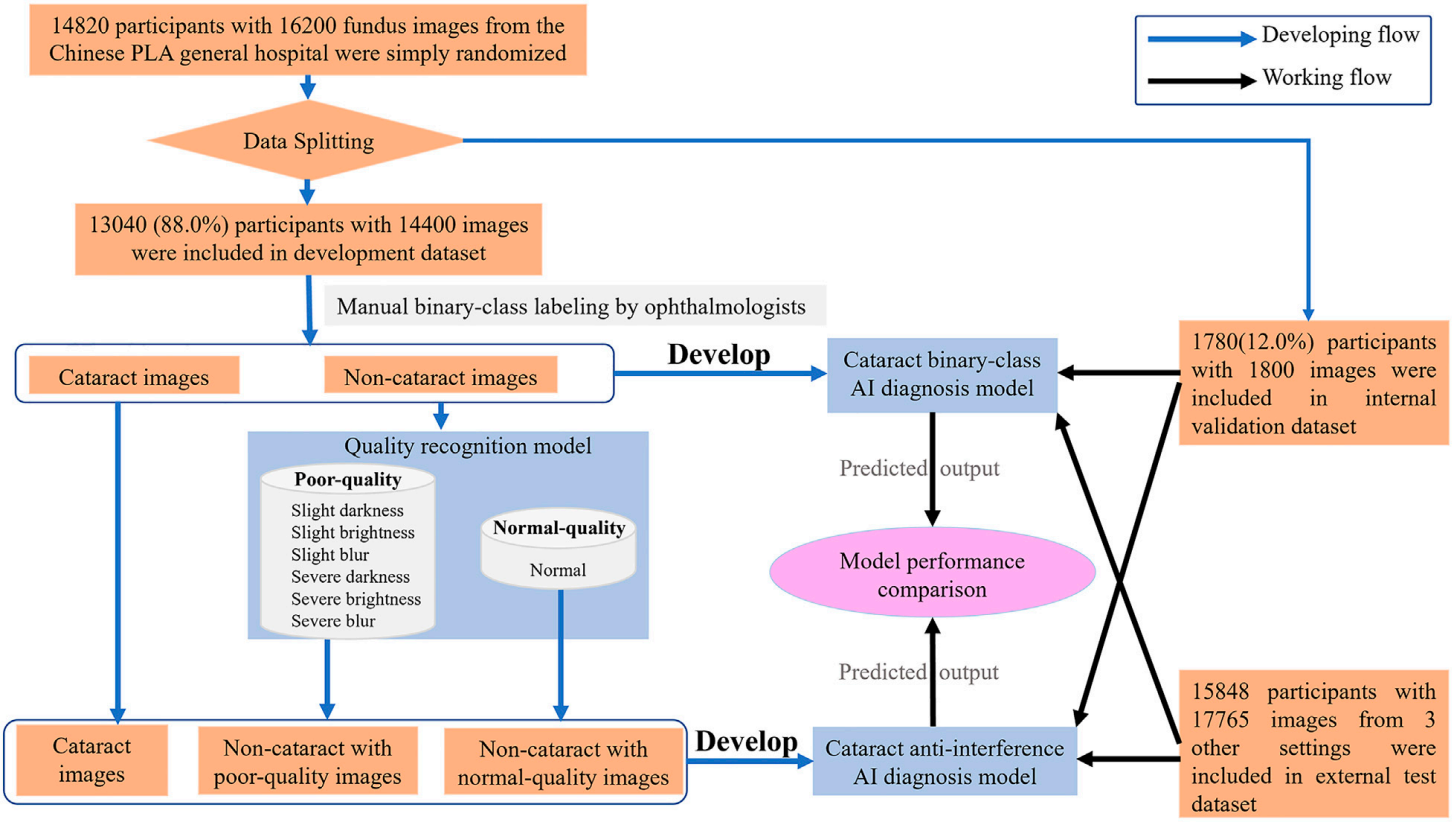
Flow chart describing the datasets and methods used for our artificial intelligence model.

In the study, the dataset which included 14,820 participants and 16,200 fundus images of cataract and noncataract was retrospectively derived from the Chinese PLA general hospital from September 2018 to May 2021. The participants’ basic information (age, sex), brief medical history with related examinations such as slit lamp images, and fundus images were anonymized and acquired from the hospital information system. The fundus images were excluded from the study if the participants had congenital cataract, intraocular lens, aphakic eye, severe eye trauma, or corneal opacity. The 14,820 participants with fundus images and medical information were simply randomized into the development dataset (88%) and internal validation dataset (12%). The 16,200 fundus images were split randomly into mutually exclusive sets for training dataset (development dataset) and internal testing dataset (internal validation dataset) of the AI model at an 8:1 ratio. To validate the availability of the antiinterference AI model in a real-world scenario, 17,765 fundus images of 15,848 participants were prospectively collected (from June 2021 to December 2021) from three real-world settings (i.e., iKang Guobin Healthcare Group Co., Ltd., Shanghai Shibei Hospital of Jing’an District and Beijing Tsinghua Changgung Hospital) as external test dataset (Shown in Figure 2). In our study, three different nonmydriatic fundus cameras (Canon, Syseye, and Topcon) were used. All fundus images were macula and optic disk-centered 45-color fundus photographs.

Each image was independently evaluated and labeled by two experienced ophthalmologists, and a third ophthalmologist was consulted in case of disagreement (shown in Figure 1A). The medical information corresponding to each recruited fundus image was provided to the ophthalmologists to improve diagnostic accuracy. The fundus images were labeled into two categories, namely, cataract and noncataract.

### Introduction of the Artificial Intelligence Model Training and Validation

For our model training stage, the model consisted of two AI submodules, a quality recognition model for cataract labeling and a CNN-based model for cataract classification, which were both trained on the development dataset. The AI model used in this study was based on deep neural network architecture. In order to obtain an effective model for real clinical usage, the whole model learning process needed to involve two steps, model training and model validation. In the model training stage, the goal was to train an AI model to fit the training data points and be competent for the specific problem. One common approach was to let the model learn with labeled data sets that were annotated by professionals. The labels were used as supervisory signals to guide models to have better capabilities to recognize cataracts. The validation stage aimed to predict the results of the input images and validate the performance of the model. During the validation stage, labels were not available during the prediction and were used to measure the performance after the model output its predictions. In our experiment, the model was tested in the internal and external validation dataset, and the results output by the AI model were then compared with the ground truth to evaluate the performance of the model.

### Quality Recognition Stage

In the practical scene, the performance of the cataract AI classification model could be largely affected by the image quality, especially when the cataract images are easily confused with poor-quality images of noncataract; thus, the correct distinction between the two is crucial for the performance of the following cataract AI recognition model. Therefore, in the first stage, we built a quality recognition model that aimed to assess the fundus images according to the image quality and generated the three-category cataract labeling for the next step.

Based on this situation, we proposed a label-based method to better distinguish cataract from noncataract with suspected fundus images using the quality recognition model. The quality recognition model was trained with a seven-category task, including normal, slight darkness, slight brightness, slight blur, severe darkness, severe brightness, and severe blur. This quality recognition model is the quality control tool for fundus images developed by Hu et al. (2021) in Airdoc company in 2019, which can be widely used in many retinal disease recognition tasks on fundus images. The model consists of two steps, the first one uses a generative adversarial network to determine whether the images are fundus images, and the second step is applied to output the probabilities of each quality grade (seven in total) of images. By combining the fundus image recognition model with the image quality classification network model, the overall accuracy can be improved by filtering out the interference from nonfundus images while obtaining a more accurate fundus image quality classification. The area under the curve (AUC) of the quality model was 99%, which was suitable for quality recognition of noncataract images; thus, we have applied this method to our work. Moreover, we employed the model to generate pseudo labels for noncataract, distinguishing between normal quality and poor quality. Therefore, by labeling the categories (slight darkness, slight brightness, slight blur, severe darkness, severe brightness, and severe blur) as poor-quality noncataract class, the original annotations were further refined into three categories (cataract, noncataract with normal-quality images, and noncataract with poor-quality images), which would give guidance to our cataract AI diagnosis model on the aspect of the label, so that the AI model could improve the performance of cataract classification (shown in Figure 1B).

The quality recognition model in our method was mainly based on Inception-Resnet pretrained on ImageNet. During training, the model was optimized with the cross-entropy loss, which was defined as follows.

$$L=-\frac{1}{N}\sum_{n=1}^{N}\sum_{i=1}^{C}y_{i}^{\left(n\right)}log\left(p_{i}^{\left(n\right)}\right)\;$$ (1)

N was the batch size, $y_{i}^{\left(n\right)}$ represented the label in of sample n (if the sample n belonged to class i , the value of $y_{i}^{\left(n\right)}$ was equal to 1; otherwise, $y_{i}^{\left(n\right)}=0$ ), C was the class number, and $p_{i}^{\left(n\right)}$ meant the predicted probability in class i of sample n. In the quality recognition stage, for hyperparameters configuration, the model of seven-class classification was trained for 200 epochs with a batch size of 24, a dropout of 0.5, and an initial learning rate of 0.0001. In addition, we used stochastic gradient descent as an optimizer, SoftMax as the last activation function, and Pytorch ReduceLRonPlateau with factor 0.2 and patience 6 as a scheduler.

### Cataract Classification Stage

Based on the quality recognition model, an antiinterference AI classification model with a CNN was trained to predict the label of images (cataract, noncataract with normal-quality images, and noncataract with poor-quality images).

CNN is a kind of feedforward neural network with depth structure and convolution calculation (Sun et al., 2019). It is one of the representative algorithms of deep learning. Because of its depth and massive layers, CNN has huge representation power to learn visual features of ophthalmic diseases and discriminate them effectively. The structure of the cataract AI classification model was mainly based on Inception-Resnet pretrained on ImageNet (Szegedy et al., 2017). As shown in Figure 1C, a CNN consisted of several convolution layers, a pooling layer, and a fully connected layer. The convolution and pooling layers cooperated to form multiple convolution groups, extract features layer by layer, and finally complete the classification through the fully connected layer. More specifically, random rotation, a data argumentation method, was applied in the data preprocessing stage, and then, each image was resized to 300px×300px as input into the model. After CNN analysis, the model output three values in the range 0–1, each representing the probability of the corresponding category for each image. At last, the category corresponding to the largest value was selected using the model, which was the predicted classification result of the image.

**FIGURE 3:**
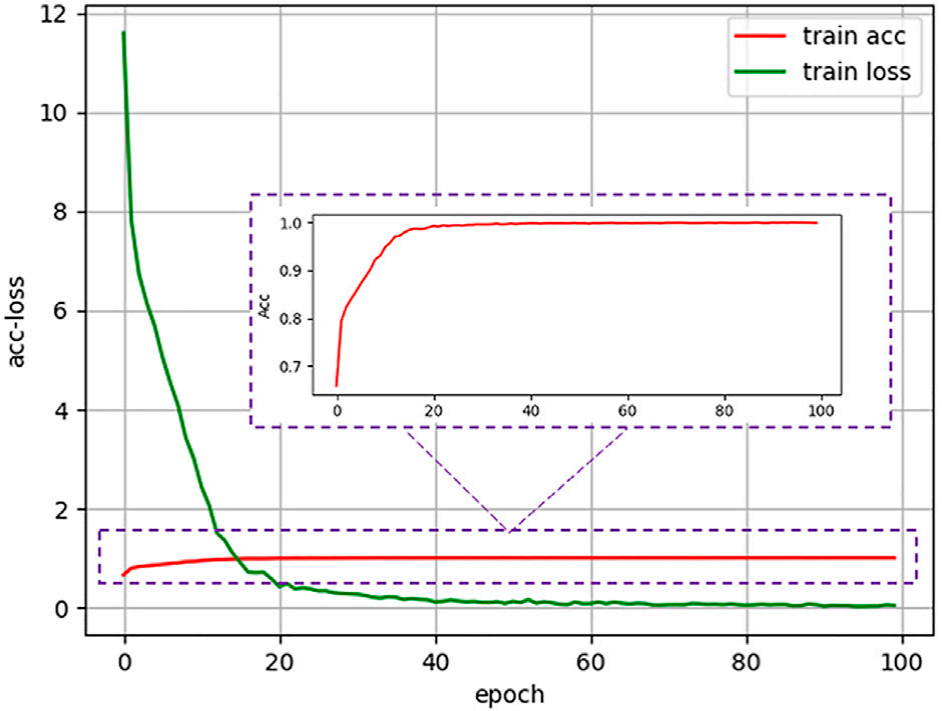
Accuracy and loss curve of antiinterference cataract artificial intelligence diagnosis model in the training process.

During the training stage, we used the fundus images from the development dataset as input to train the models. After one hundred training epochs, a cataract classification model can be obtained, and the accuracy and loss curve during the training process were shown in Figure 3. As for implementation details, we trained the network on our dataset for 100 epochs with a batch size of 24. Stochastic gradient descent with a momentum of 0.9 and a weight decay of $10^{-4}$ was used as the optimizer. In addition, the initial learning rate was set as 0.0001, and SoftMax was chosen as the last activation function. In the internal validation and external test stage, fundus images were mixed and used as input, and the cataract AI classification model could predict and output classification labels directly.

To verify the robustness of our proposed method, we set up a control experiment, in which the cataract diagnosis model was trained on the binary-class labels, including normal and cataract two categories. In addition, the setting of the control experiment was consistent with the proposed method. Then, we tested the performance of the cataract binary-class AI model in the control experiment and the antiinterference cataract AI diagnosis model concerning standard diagnosis based on ophthalmologists’ evaluation in the same dataset (internal validation set and external test dataset, which contained a certain amount of poor-quality fundus images). Furthermore, we tested the performance of the antiinterference model in the external test dataset.

### Statistical Analysis

The indices used for evaluation were calculated using the accuracy of the formula (ACC) = (TP + TN)/(TP + TN + FP + FN), sensitivity (SEN) = TP/(TP + FN), and specificity (SPE) = TN/(TN + FP), where TP is true positive, TN is true negative, FP is false positive, and FN is false negative. Asymptotic two-sided 95% CIs presented as the AUC and were calculated by using bootstrap analysis with 100,000 random seed sampling. Receiver operating characteristic curves were created using the R statistical package, V.3.2.4. To visualize the decision ways of the model, we applied the Grad-CAM to generate heatmaps.

## Results

### Basic Information of Recruited Cases

A total of 30,668 participants with 33,965 fundus images were recruited for this study (Table 1). Among them, 15,804 (51.53%) are male and 14,864 (48.47%) are female. In the development, internal validation, and external test dataset, the participants in different sex groups were relatively evenly distributed, respectively.

**TABLE 1:** Characteristics of the development, internal validation, and external test dataset.

Characteristics	Development dataset		Internal validation dataset		External test dataset	
	Male	Female	Male	Female	Male	Female
No. of participants	6,745	6,295	947	833	8,112	7,736
Age	53.00 ± 15.09	52.81 ± 14.80	52.16 ± 14.96	53.89 ± 14.87	52.92 ± 15.19	53.03 ± 15.02
No. of images	7,498	6,902	960	840	9,201	8,564
Cataract	2,298	2,502	273	327	2,831	3,166
Noncataract with normal-quality images	2,469	2,331	326	274	3,008	2,992
Noncataract with poor-quality images	2,731	2,069	361	239	3,362	2,406

### Evaluation of Cataract Artificial Intelligence Diagnosis Model Based on the Original Binary-Class Labels

**FIGURE 4:**
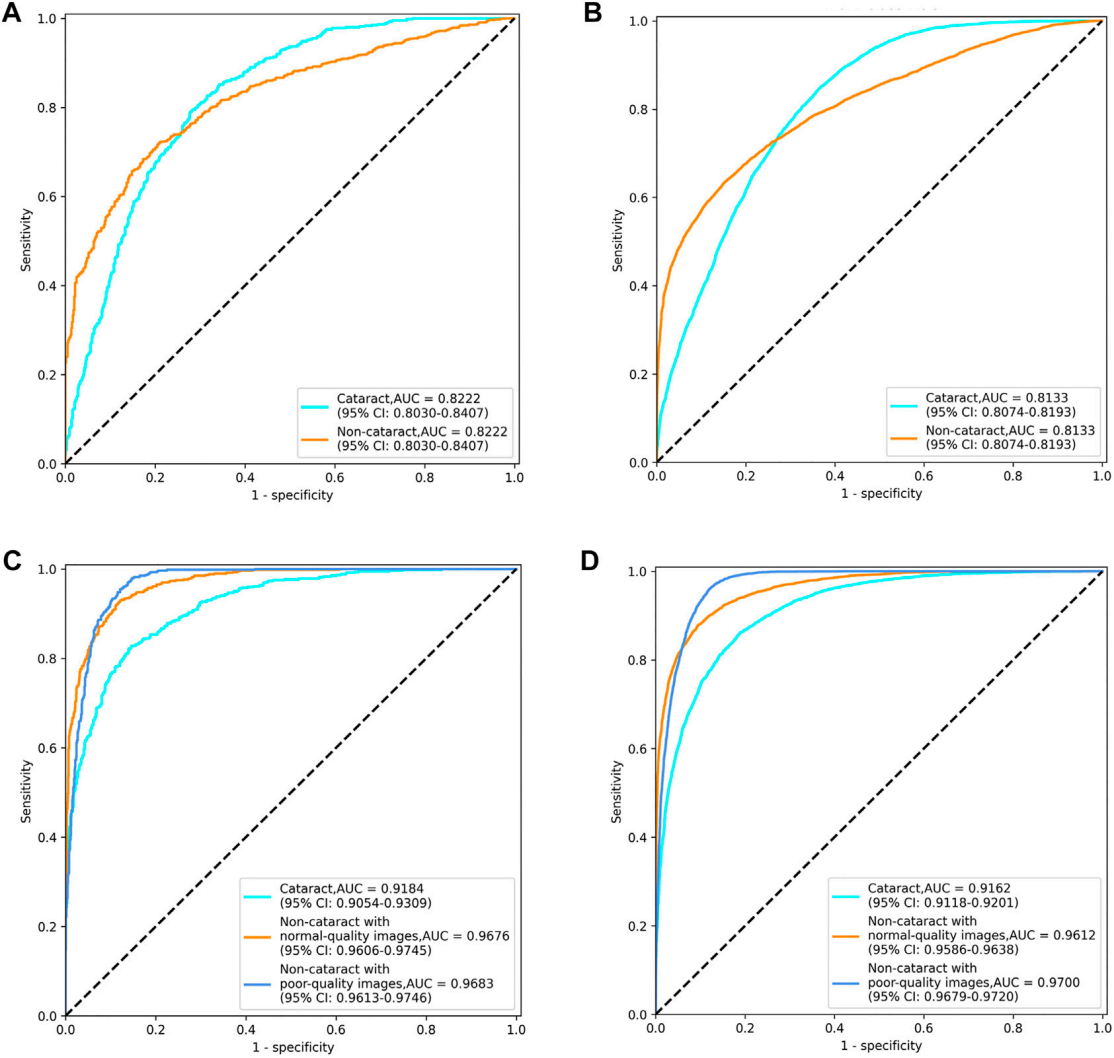
Receiver operating characteristic curves of the two cataract artificial intelligence diagnosis models. **(A)** binary-class model in the internal validation dataset. **(B)** binary-class model in the external test dataset. **(C)** antiinterference model in the internal validation dataset. **(D)** antiinterference model in the external test dataset.

**FIGURE 5:**
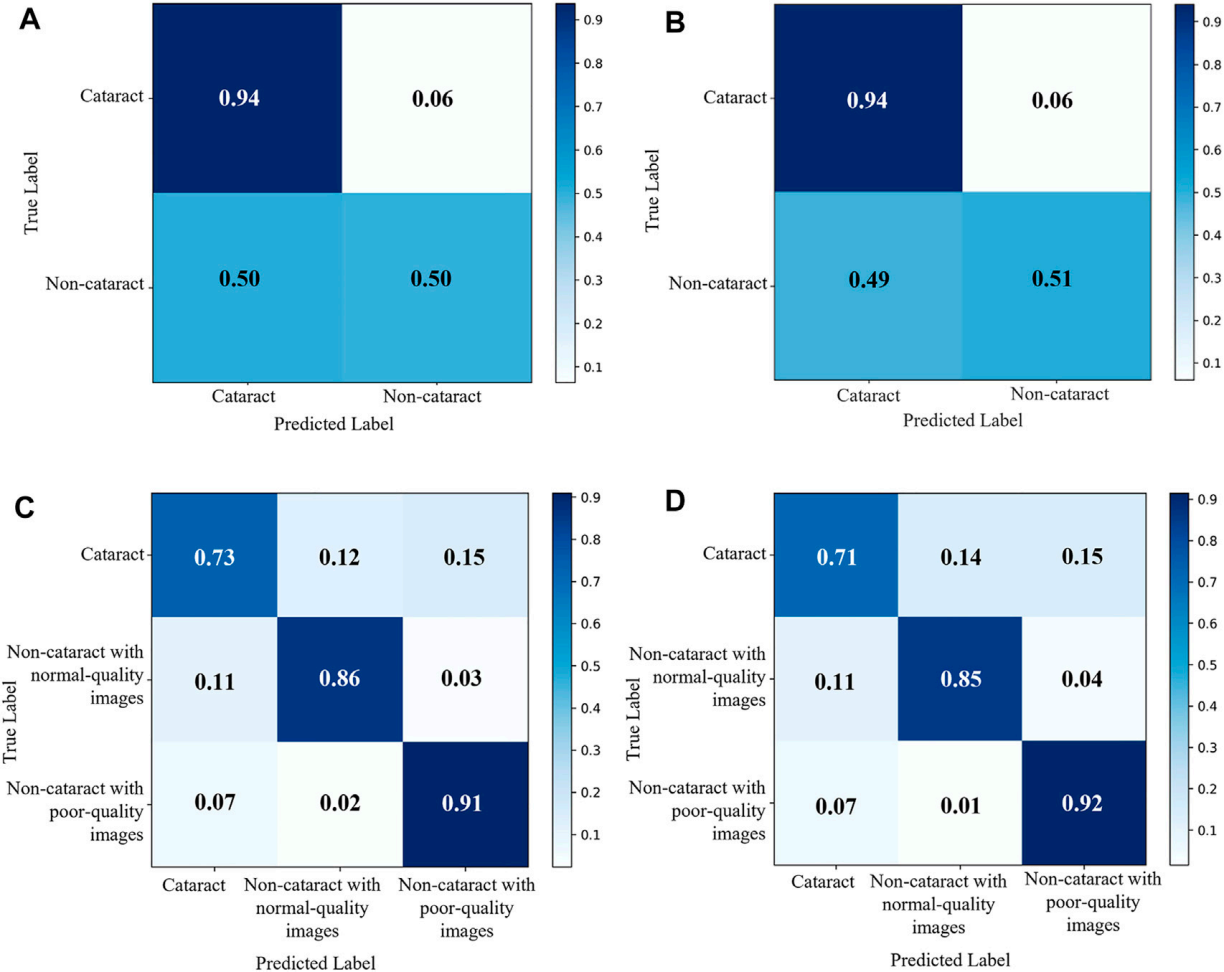
Confusion matrix of the two cataract artificial intelligence diagnosis models. **(A)** binary-class model in the internal validation dataset. **(B)** binary-class model in the external test dataset. **(C)** antiinterference model in the internal validation dataset. **(D)** Antiinterference model in the external test dataset.

The diagnostic performance of the cataract AI diagnosis model in the control experiment on the internal validation dataset was shown in Table 2; Figure 4A, Figure5A. The model determined the diagnosis of cataracts with an AUC of 82.22% and an ACC of 64.33%. From the confusion matrix, shown in Figure 5A, we could see that there were many noncataract images were mistakenly classified as cataract due to the interference of poor-quality fundus images; thus, the performance of cataract recognition degraded to a certain degree.

**TABLE 2:** erformance of the two cataract artificial intelligence diagnosis models in the internal validation dataset.

Classification	AUC (%)	ACC (%)	SEN (%)	SPE (%)
Binary-class model				
Cataract	82.22	64.33	93.67	49.67
Anti-interference model				
Cataract	91.84	85.06	73.17	90.75
Noncataract with normal-quality images	96.76	90.44	85.67	91.97
Noncataract with poor-quality images	96.83	91.06	91.00	89.91

AUC, area under the curve; ACC, accuracy; SEN, sensitivity; SPE, specificity.

For the results of the binary-class model in the external test dataset, the receiver operating characteristic curve and confusion matrix diagram were exhibited in Table 3; Figures 4B, 5B. The AUC and ACC in the external test dataset were 81.33% and 65.34%. In addition, similar to the performance in the internal validation dataset, the performance of the model was also affected to some extent by poor-quality images.

**TABLE 3:** erformance of the two cataract artificial intelligence diagnosis models in the external test dataset.

Classification	AUC (%)	ACC (%)	SEN (%)	SPE (%)
Binary-class model				
Cataract	81.33	65.34	94.03	50.72
Anti-interference model				
Cataract	91.62	84.37	71.20	90.84
Noncataract with normal-quality images	96.12	89.82	85.37	91.11
Noncataract with poor-quality images	97.00	90.97	91.50	89.39

AUC, area under the curve; ACC, accuracy; SEN, sensitivity; SPE, specificity.

### Evaluation of Antiinterference Cataract Artificial Intelligence Diagnosis Model Based on Three Categories of Labels

Receiver operating characteristic curves and confusion matrix diagrams of the antiinterference cataract AI diagnosis model was shown in Figures 4C,D; Figures 5C,D. The model determined diagnosis of cataract, noncataract with normal-quality images, or noncataract with poor-quality images with AUC of 91.84%, 96.76%, and 96.83% in the internal validation dataset, respectively (Table 2). Compared with the model that was trained on the binary-class label, the antiinterference cataract model improved its performance by 10%.

The model determined diagnosis of cataract, noncataract with normal-quality images, or noncataract with poor-quality images with AUCs of 91.62%, 96.12%, and 97.00% in the external test dataset, respectively (Table 3).

### Heatmap Visualization

**FIGURE 6:**
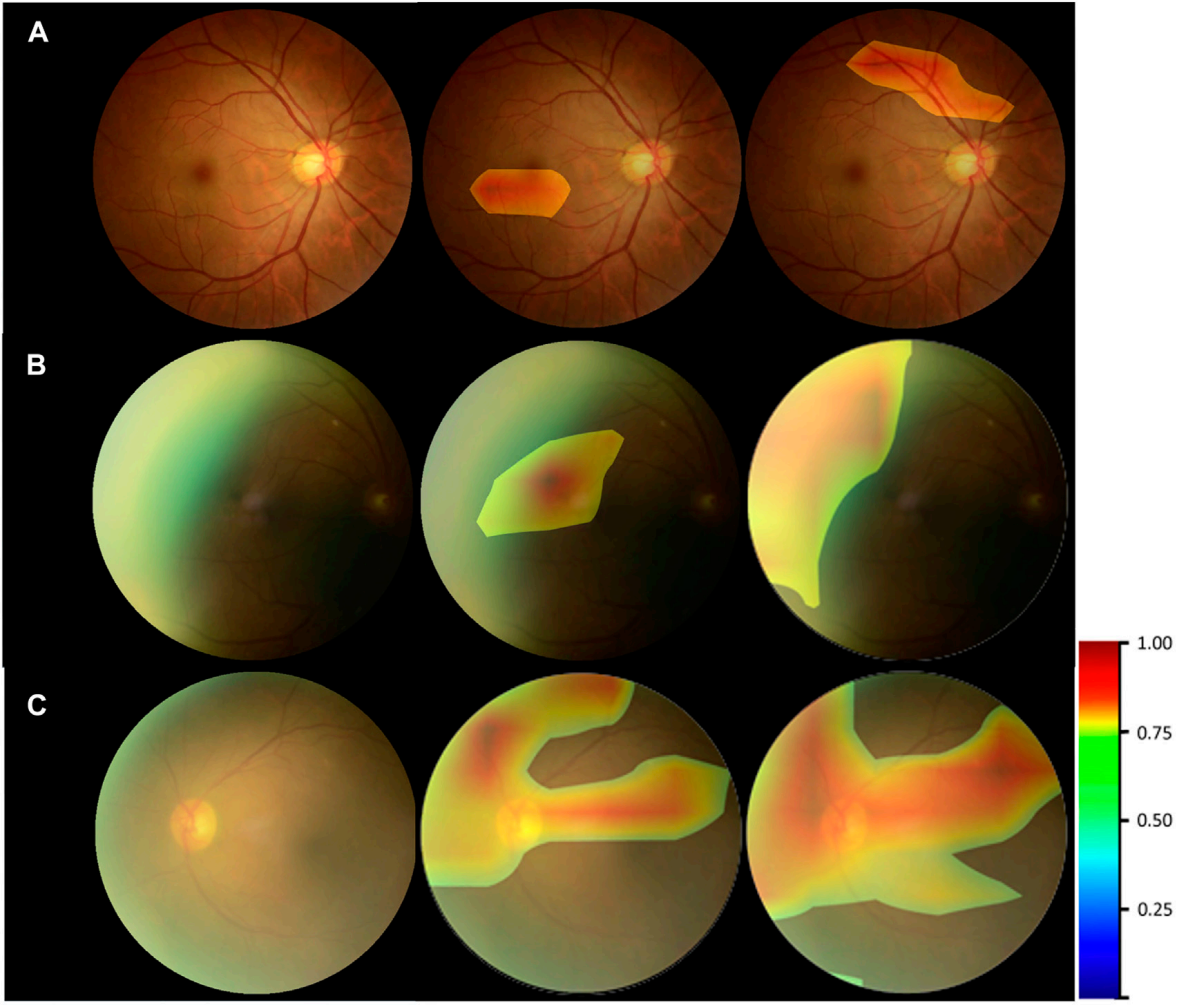
Heatmap visualization examples. Left column: the original fundus images; middle column: general heatmap of antiinterference method; right column: general heatmap of control experiment. **(A)** noncataract with normal-quality image. **(B)** noncataract with poor-quality image. **(C)** cataract.

Figure 6 provided the visual feature map both in our proposed method and control experiment. We analyzed the heatmaps in two aspects. First, by comparing our approach with the control experiment, it could be seen that for the cataract and noncataract with normal-quality images, the attention regions of both methods were similar, with cataract focusing on the blurred areas, optic disk, and great vessels and noncataract with normal-quality image concerning the small and medium vessels (Figure 6A). However, for the noncataract with poor-quality images, our proposed method focused more on the important part, which showed a better ability to distinguish cataracts with the suspected cataract images. For instance, in Figure 6B, a noncataract with poor-quality images was misclassified by the control experiment as the cataract class because it expressed a similar yellow margin of the peripheral area with cataract images, which was due to the light leakage from the device. However, our method paid more attention to the light change and darker area rather than the blur and suspected cataract part. Second, comparing the attention area that our model focused on and the cataract criterion of professional doctors, they had the consistent regulations that were based on the blur degree of whole fundus images and the visibility of the vessels and optic disk to determine the cataract (Figure 6C). Therefore, through the visual analysis, it showed that our method had a better ability to diagnose cataracts than the control experiment.

## Discussion

Cataract is the most common cause of blindness worldwide with the characteristic of lenticular opacity. With the rapid development of AI in medicine, the AI model can effectively identify the details such as blood vessels in the fundus images, which makes it feasible for automatic diagnosis through the fundus images. Several studies have been reported using an AI model based on fundus images for cataract diagnosis. In the fundus images taken from subjects with mild cataracts, small retinal vessels are visible but slightly blurred. With the aggravation of cataracts, more structures will be invisible until nothing can be seen.

In the previous study, the cataract AI model developed from a single learning model to multiple learning models and improved the prediction accuracy substantially. Yang et al. (2013) built a neural network classifier that consists of three parts: preprocessing, feature extraction, and classifier construction. Imran et al. (2021) proposed a novel hybrid convolutional and recurrent neural network for cataract classification and increased an average accuracy of 97.39% for four-class cataract classification. Yadav and Yadav (2022) studied computer-aided cataract detection and grading by extracting and fusing features and integrating the predictions through machine learning methods, which achieved 96.25% four-class classification accuracy. The six-level classification of cataracts could enable ophthalmologists to accurately understand the patient’s condition, and the average accuracy of the six-grading model was up to 92.66%. However, all above this, the fundus images with good quality are the key points (Zhang et al., 2019). Here, we reported that the accuracy of the antiinterference model was approximately 83%, which is lower than that in the previous study. This may be due to our inclusion of fundus images with poor quality in the study, resulting in complicated interference.

Studies have shown that the fuzziness and scanning quality index of fundus images are related to cataract AI identification and grading (Xu et al., 2010; Chen et al., 2021). Poor-quality images can greatly affect the accuracy of the results (Zhang et al., 2019). The common limitation of previous studies is that there are high requirements for the quality of fundus images. However, in the actual cataract screening scenario, it is difficult to ensure that all of the fundus images meet the quality requirements because of the uneven technical level of operators and inadequate cooperation of patients. The interference of poor-quality images caused by shooting encountered in the research has not been solved.

In the study, we developed and validated an antiinterference cataract diagnosis model that can identify quality problems of fundus images to be better applied in the real world. The model that can achieve accurate cataract recognition mainly included a quality recognition model for adjusting cataract labels and a CNN-based model for cataract classification. The quality recognition model aimed to distinguish between cataract images and noncataract with poor-quality images that are easy to be misclassified by converting the original binary-class label into a triplet. Then, we trained cataract diagnosis models based on dichotomy and trichotomy respectively and compared their performance. According to the results of this study, we found that the antiinterference cataract model improved the performance by 10% compared with the model that was trained on the binary-class label; hence, the quality recognition model can enhance the robustness of the cataract AI diagnosis model.

In the primary medical facilities of low- and middle-income countries, a cataract cannot be diagnosed until it develops to an advanced stage and even the symptoms can be observed with the naked eye. Our cataract AI diagnosis model is helpful for the early detection of cataracts. If participants are diagnosed with cataracts using the AI model, they need to go to the hospital and follow the doctor’s advice for further examination, such as slit lamp and ophthalmic B-type ultrasound. If the output of the AI model is noncataract with poor-quality images, it would be best to retake new fundus images with normal quality or after mydriasis as early as possible. Assuming that the output of the AI model is noncataract with normal-quality images, the participants need to be retested in 12 months.

Considering that the dataset used for internal validation has similar characteristics to the dataset used for model training, it may lead to better accuracy and stability of the research results than the real situation. Therefore, further external validation was conducted, and our proposed model showed good performance. In the future, a larger sample size database will help to optimize the antiinterference cataract model (Chen et al., 2021).

In conclusion, we developed an AI model for antiinterference cataract automatic diagnosis based on fundus images. The antiinterference model achieved cataract diagnosis with high accuracy even in the presence of poor-quality image interference, allowing the model to provide cataract screening and help the government formulate a more accurate aid policy. In recent 10 years, the application of AI for cataract identification and grading based on fundus images has developed rapidly. To better achieve cataract classification, further research needs to be done to develop a cataract grading AI model based on our proposed method.

## Data Availability

The raw data supporting the conclusion of this article will be made available by the authors, without undue reservation.
